# Associations of Adverse Childhood Experiences with Executive Function and Brain-Derived Neurotrophic Factor

**DOI:** 10.1093/geroni/igab046.3671

**Published:** 2021-12-17

**Authors:** Cindy Tsotsoros, Madison Stout, Misty Hawkins

**Affiliations:** 1 Oklahoma State University, Stillwater, Oklahoma, United States; 2 Oklahoma State University, Oklahoma State University, Oklahoma, United States

## Abstract

Adverse childhood experiences (ACEs) may predict markers of neurocognitive performance (i.e., executive function; EF) and brain health/plasticity (i.e., brain-derived neurotropic factor; BDNF). This pilot examined the magnitude of effects between: 1) ACES and EF performance, 2) ACEs and BDNF levels, and 3) EF performance and BDNF levels. We hypothesize that higher ACEs will be associated with poorer EF scores and lower BNDF levels and that lower EF scores will be associated with lower BDNF levels. Given the pilot nature of the study, an emphasis is placed on effect size vs. significance. Participants were 36 middle-aged women enrolled in the NICE SPACES trial (age=31.4 years, BMI=34.2, racially minoritized=37.9%). ACES were quantified using the 10-item Adverse Childhood Experiences Scale. EF was measured using the fluid cognition composite from the NIH Toolbox – Cognition Battery. BDNF was estimated using proBDNF levels estimated from serum collected via venipuncture. Higher ACEs levels were not directly associated with EF scores (b = 0.03, p = .854); but did show a meaningful negative beta coefficient with BDNF levels (b = -0.34, p = .053). EF scores and BDNF showed a positive coefficient that did not reach significance (b = .26, p = .122). In a modest pilot of middle-age women, higher ACEs were associated with lower BDNF, indicating greater adversity in childhood is linked to lower neurotrophins levels in adulthood. The lower BDNF levels may help explain poorer performance on cognitive tasks. Larger follow-up studies in more powered samples are warranted given the size of detected coefficients.

